# Antimicrobial activity of selected South African medicinal plants

**DOI:** 10.1186/1472-6882-12-74

**Published:** 2012-06-14

**Authors:** Trine R H Nielsen, Victor Kuete, Anna K Jäger, Jacobus J Marion Meyer, Namrita Lall

**Affiliations:** 1 Department of Medicinal Chemistry Faculty of Pharmaceutical Sciences, University of Copenhagen, Copenhagen, Denmark; 2 Department of Biochemistry, Faculty of science, University of Dschang, P.O. Box 67, Dschang, Cameroon; 3 Department of Plant Science, Faculty of Agricultural and Biological Science, Pretoria 0002, South Africa

## Abstract

**Background:**

Nearly 3,000 plant species are used as medicines in South Africa, with approximately 350 species forming the most commonly traded and used medicinal plants. In the present study, twelve South African medicinal plants were selected and tested for their antimicrobial activities against eight microbial species belonging to fungi, Mycobacteria, Gram-positive and Gram-negative bacteria.

**Methods:**

The radiometric respiratory technique using the BACTEC 460 system was used for susceptibility testing against *Mycobacterium tuberculosis,* and the liquid micro-broth dilution was used for other antimicrobial assays.

**Results:**

The results of the minimal inhibitory concentration (MIC) determinations indicated that the methanol extracts from *Acacia karoo, Erythrophleum lasianthum* and *Salvia africana* were able to prevent the growth of all the tested microorganisms. All other samples showed selective activities. MIC values below 100 μg/ml were recorded with *A. karoo*, *C. dentate*, *E. lasianthum*, *P. obligun* and *S. africana* on at least one of the nine tested microorganisms. The best activity (MIC value of 39.06 μg/ml) was noted with *S. africana* against *E. coli, S. aureus* and *M. audouinii*, and *Knowltonia vesitoria* against *M. tuberculosis.*

**Conclusion:**

The overall results of the present work provide baseline information for the possible use of the studied South African plant extracts in the treatment of microbial infections.

## Background

The World Health Organization (WHO) estimates that up to 80% of the population in Africa makes use of traditional medicine as well as about 65% of the world's population [1]. Plants used in traditional medicine, also called phytomedicine are plant-derived medicines that contain chemicals, more usually, mixtures of chemical compounds that act individually or in combination on the human body to prevent disorders and to restore or maintain health [2]. With up to 19,581 indigenous species, South Africa has the richest temperate flora in the world. A minimum of 11,700 species are endemic to South Africa [3] and nearly 3,000 species are used as medicines with approximately 350 species forming the most commonly traded and used medicinal plants [4]. Hence, there is good reason to screen these traditionally used plants for new drug discovery. The ethnobotanical approach has a number of advantages when searching for new drug candidates. The plants have been ‘pre-screened’ through generations of trial and error processes occurring over hundreds or thousands of years [5]. This will result in a higher probability of producing useful therapies from a group of medicinal plants, and one can rationalize that any isolated active compounds from plants are likely to be safer than active compounds from plants with no history of human use [5,6]. The present work was therefore designed to investigate the antimicrobial activity of twelve selected South African medicinal plants, *Acacia karroo* Hayne (Mimosoideae), *Acokanthera oppositifolia* (Lam.) Codd (Apocynaceae), *Bulbine latifolia* (L.f.) Spreng var. *Latifolia* (Asphodelaceae), *Curtisia dentata* (Burm.f.) C.A.Sm. (Cornaceae), *Erythrophleum lasianthum* Corbishley (Caesalpinioideae), *Knowltonia vesicatoria* Sims (Ranunculaceae), *Ptaeroxylon obliquum* (Thunb.) Radlk. (Ptaeroxylaceae), *Salvia africana-lutea* L. (Lamiaceae), *Sansevieria hyacinthoides* Hort. ex Steud. (Dracaenaceae).

## Methods

### Plant materials and extraction

All plants were collected on the Campus area of the University of Pretoria in August 2007. The plant parts were mainly aerial parts such as leaves, stems and flowers, except for *S. hyacinthoides* where the rhizome was dug up and collected, based on the traditional uses (Table 1). The plants were then identified at the HGWJ Schweicherdt Herbarium, University of Pretoria and a voucher specimen was preserved there as well (Table 1). The air dried and powdered sample from each plant (10 g) were macerated in methanol (300 ml) for 48 h at room temperature with occasional shaking. After 48 hours the extract was filtered through a Whatmann no. 1 filter paper. The extract was then concentrated under reduced pressure to give the crude extracts. They were then kept at 4 °C until further use.

**Table 1:** Plants used in the present work

**Botanical name** **(FAMILY)**	**voucher specimen**	**Parts used, extraction yield**	**Traditional use**
*Acacia karroo* Hayne^A^ (Mimosoideae)	T. Nielsen PRU 096395	Leaves, 25.1% Stem + Bark, 13.6%	Mouth ulcers, oral thrush^E^, diarrhea, dysenteries, colic, colds^G^, other *Acacia* species: asthma, bronchitis, cough, phithisis, fever, leprosy, chest and respiratory ailments^H^.
*Acokanthera oppositifolia* (Lam.) Codd^A^ (Apocynaceae)	T. Nielsen PRU 0963388	Leaves, 27.7% Stem, 12.6%	Hunting poison^F^, snakebites^D^, abnormal menstruation, snuff for headaches, septicaemia, toothache^E^.
*Bulbine latifolia* (L.f.) Spreng var. *latifolia*^A^ (Asphodelaceae)	T. Nielsen PRU 096391	Leaves, 0.5%	Wounds, burns, skin conditions^E^, diarrhoea, dysenteries, abdominal ailments^G^, rheumatism^H^.
*Curtisia dentata* (Burm.f.) C.A.Sm.^A^ (Cornaceae)	T. Nielsen PRU 096385	Leaves, 33.7% Stem, 15.1%	Stomach ailments, diarrhea, blood purifier, afrodisiac^E^, tanning, chewing sticks^G^.
*Erythrophleum lasianthum* Corbishley^A^ (Caesalpinioideae)	No voucher specimen available *	Leaves, 41.2% Stem, 31.2%	Headaches^D^, fever^H^, *Erythrophleum* species: heart problem, dermatitis, wounds, rheumatism, syphilis, gonorrhea, leprosy, tuberculosis, bronchitis, angina, ordeal and hunting poison^F^.
*Knowltonia vesicatoria* Sims^B^ (Ranunculaceae)	T. Nielsen PRU 096390	Aerial parts, 31.4%	Tooth pain^E^, rheumatism, lumbago, colds, influenza^G^, skin tumor^H^.
*Ptaeroxylon obliquum* (Thunb.) Radlk.^A^ (Ptaeroxylaceae)	T. Nielsen PRU 096389	Leaves, 23.1%, Stem, 16.1%, Bark, 13.7%	Snuff for headaches^D^, rheumatism, arthritis^E^, heart disease, anthrax, sinusitis, lupus, warts^G^, sternutatory^H^.
*Salvia africana-lutea* L.^A^ (Lamiaceae)	T. Nielsen PRU 096396	Aerial parts, 29.2%	Colds, flu, bronchitis, abdominal and uterine troubles^E^, cough, chest troubles^G^, other *Salvia* species: night sweat tuberculosis, respiratory and pulmonary ailments, ^G,H^ .
*Sansevieria hyacinthoides* Hort. ex Steud.^B^ (Dracaenaceae)	T. Nielsen PRU 0963387	Leaves, 11.9% Rhizome, 12.7%	Earache, stomachache, toothache, hemorrhoids, ulcers, diarrhea, intestinal parasites^E^, Sansevieria species: chest diseases, cough remedy^F^.
*Searsia burchellii* (Sond. ex Engl.) Moffett^C^ (Anarcardiaceae)	T. Nielsen PRU 096386	Leaves, 24.5% Stem, 9.4%	Chest colds^G^, *Rhus (Searsia)* species: colds, cough, expectorant, febrifuge, pulmonary ailments, tuberculosis^H^.
*Typha capensis* Rohrb.^B^ (Typhaceae)	T. Nielsen PRU 096394	Leaves, 17.0%	Fertility, potency, circulation, delivery, venereal diseases, dysentery, diarrhea, enteritis, blood purifier^E^, gonorrhea, pulmonary ailments^H^.
*Zantedeschia aethiopica* (L.) Spreng.^A^ (Araceae)	T. Nielsen PRU 096392	Leaves + Stem, 15.2%	Wounds, sores, boils^E,G^, insect bites, gout, rheumatism^G,H^.

A: A checklist of South African plants [3]; B: The International Plants Names Index [7] C: Moffett [8]; D: Trees of Southern Africa [9]; E: People’s Plants [2], F: African Ethnobotany [10]; G: The Medicinal and Poisonous Plants of Southern and Eastern Africa [11]; H: CRC Ethnobotany Desk Reference [12].

* No reproductive parts (flowers, fruits) could be collected from this plant; consequently no herbarium specimen is available.

### Chemicals for antimicrobial assay

Nystatin (Maneesh Pharmaceutic PVT) for fungi, ciprofloxacin and isoniazid (INH) (Sigma-Aldrich, Johannesburg, South Africa) for *Mycobacterium smegmatis and M. tuberculosis* respectively and gentamicin (Jinling Pharmaceutic Group corp.) for other bacteria were used as reference antibiotics (RA).

### Microorganisms and culture media

The tested organisms included *Mycobacterium smegmatis* ATCC 700084 [obtained from South African Medical Research Council, Pretoria and authenticated by Professor Fanus Venter, Department of Microbiology and Plants Pathology, University of Pretoria], drug-susceptible strain of *Mycobacterium tuberculosis* H37Rv ATCC 27294 (America Type Culture Collection, Rockville, MD, USA), Methicillin-resistant *Staphylococcus aureus* (MRSA, LMP805) (Gram-positive bacterium), four Gram-negative bacteria namely β-lactamase positive (βL^+^) *Escherichia coli* (βL^+^EC, LMP701), Ampicillin-resistant *Klebsiella pneumoniae* (ARKP, LMP803), Carbenicillin-resistant *Pseudomonas aeruginosa* (CRPA, LMP804), Chloramphenicol-resistant *Citrobacter* (CRCF, LMP802) and two fungi namely *Candida albicans* (*C. albicans* LMP709U) and *Microsporum audouinii* (*M. audouinii*, LMP725D). *M. smegmatis* was cultured on Middlebrook 7 H11 agar and allowed to grow for 24 h. *M. tuberculosis* was plated on Löwenstein–Jensen medium and allowed to grow for 3–4 weeks at 37 °C. The 7 H9 broth was used to determine the minimal inhibition concentration (MIC) of the test samples on *M. smegmatis* and *M. tuberculosis*. Other microbial species were clinical isolates from Yaoundé General Hospital (Cameroon). Their identity was confirmed before use at the Laboratory of Applied Microbiology and Molecular Pharmacology (LMP) (Faculty of Science, University of Yaoundé I) by culturing on the specific media followed by biochemical test using API system as previously reported [13]. All the studied microorganisms were maintained on agar slant at 4 °C at the LMP. These strains were sub-cultured on a fresh appropriate agar plate 24 hours prior to any antimicrobial test. The Mueller Hinton broth (MHB) was used to determine the MIC of all samples against the tested pathogens. The MHB and Mueller Hinton Agar (MHA) were used to determine the minimal microbicidal concentration (MMC) of the active samples.

### Microplate susceptibility testing against M. Smegmatis

All extracts were tested against *M. smegmatis* using the microplate dilution method. The MIC, MBC and bacteria preparation were performed in 96-well microplates as previously described [14,15]. The crude extracts were dissolved in 10% dimethylsulfoxide (DMSO) in sterile 7 H9 broth to obtain a stock concentration of 10 mg/ml. Serial two-fold dilutions of each sample to be evaluated were made with 7 H9 broth to yield volumes of 100 μl/well with final concentrations ranging from 19.53–2500 μg/ml. Ciprofloxacin served as the positive drug control. One hundred microlitre of *M. smegmatis* (0.2 log-phase, yielding 1.5 × 10^6^ CFU/ml) were also added to each well containing the samples. The solvent control, DMSO at 2.5% or less in each well did not show inhibitory effects on the growth of *M. smegmatis*. Tests were done in triplicates. The cultured microplates were sealed with parafilm and incubated at 37 °C for 24 h. The MIC was detected following addition (40 μl) of 0.2 mg/ml *p*-iodonitrotetrazolium chloride (INT, Sigma-Aldrich) and incubated at 37 °C for 30 min [16,17]. Viable bacteria reduced the yellow dye to a pink color. MIC was defined as the lowest sample concentration that prevented this change and exhibited complete inhibition of bacterial growth. The MBC was determined by adding 50 μl aliquots of the preparations (without INT), which did not show any growth after incubation during MIC assays, to 150 μl of 7 H9 broth. These preparations were incubated at 37 °C for 48 h. The MBC was regarded as the lowest concentration of extract, which did not produce a color change after addition of INT as above mentioned.

### Antitubercular rapid radiometric assay using mycobacterium tuberculosis

The radiometric respiratory technique using the BACTEC 460 system (Becton Dickinson Diagnostic Instrument, Sparks, MD) was used for susceptibility testing against *M . tuberculosis* as described previously [17-19] with some modifications. Solutions of all the samples were prepared in Dimethylsulfoxide (DMSO) to obtain a stock concentration. Control experiments showed that a final concentration of DMSO at 1% in the medium had no adverse effect on the growth of *M. tuberculosis*. The primary drug INH served as drug-control. A homogenous culture (0.1 ml of *M. tuberculosis*, yielding 1 × 10^4^ to 1 × 10^5^ CFU/ml) was inoculated in the vials containing samples as well as control vials [20]. Three sample-free vials were used as controls (medium + 1%DMSO); two vials (V1) were inoculated in the same way as the vials containing the sample, and one (V2) was inoculated with a 1:100 dilution of the inoculum (1:100 control) to produce an initial concentration representing 1% of the bacterial population (1 × 10^2^ to 1 × 10^3^ CFU/ml). *Mycobacterium* growing in 7 H12 medium containing ^14^ C labeled substrate (palmitic acid) uses the substrate and produced ^14^CO_2_. The amount of ^14^CO_2_ detected is expressed in terms of the growth index (GI) [21]. Vials were incubated at 37 °C and each was assayed everyday to measure the GI. On the Bactec TB-460 system, the GI is recorded on a scale of 0–999. Growth is detected when GI ≥ 20. Then the GI value of 10 represents the growth of 1% of the bacterial population, once the inoculum control reach 999. Therefore, the MIC was considered as the lowest concentration inhibiting more than 99% of the initial bacterial population. This was taken as the lowest concentration of the sample with recorded Δ*G* < 10. Δ*G* = *Gx* − *G*0; *Gx* being the GI of the treated medium on the day that control inoculum vial reach 999, *G*0 the GI at the first day of reading and Δ*G*, the difference between *Gx* and *G0*[22].

### Antimicrobial assay on gram-positive, gram-negative bacteria and fungi

The MICs of crude extracts and reference antibiotics (RA) (gentamicin for bacteria and nystatin for fungi) were determined as follows: the test sample was first of all dissolved in dimethylsulfoxide (DMSO). The solution obtained was added to MHB to give a final concentration of 2500 μg/ml for the crude extracts and 78.12 for RA. This was serially diluted two fold to obtain concentration ranges of 19.53 to 2500 μg/ml for the crude extracts and 0.31 to 78.12 μg/ml for RA. One hundred microlitre of each concentration were added to each well (96- wells microplate) containing 95 μl of NBGP and 5 μl of inoculum (standardized at 1.5 10^6^ CFU/ml by adjusting the optical density to 0.1 at 600 nm SHIMADZU UV-120-01 spectrophotometer) [23,24]. The final concentration of DMSO in the well was less than 1% (preliminary analyses with 1% (v/v) DMSO do not affect the growth of the test organisms). The negative control well consisted of 195 μl of MHB and 5 μl of the standard inoculum [23,24]. The plates were covered with a sterile plate sealer, then agitated to mix the contents of the wells using a plate shaker and incubated at 37 °C for 24 hours. The assay was repeated thrice. The effect of samples was detected following addition (40 μl) of 0.2 mg/ml *p*-iodonitrotetrazolium chloride and incubated at 37 °C for 30 min [25]. MIC and MMC were determined as above [23,24].

## Results and discussion

The results of the antimicrobial activity of the studied plant extracts are summarized in Table 2 and 3. The results of the MIC determinations (Table 2) showed that the extracts from *A. karoo, E. lasianthum stem* and *S. africana* were able to prevent the growth of all the tested microorganisms. All other samples showed selective activities. Phytochemicals are routinely classified as antimicrobials on the basis of susceptibility tests that produce MIC in the range of 100 to 1000 µg/ml [25]. Activity is considered to be significant if MIC values are below 100 μg/ml for crude extract and moderate when 100 < MIC < 625 μg/ml [26,27]. Therefore, the activity recorded with *A. karoo* (leaves extract on *C. albicans, M. audouinii* and stem extract on *M. audouinii* and *K. pneumoniae*), *C. dentate* (leaves extract on *C. albicans* and stem bark extract on *C. albicans* and *S. aureus*), *E. lasianthum* on *S. aureusP. obligun* bark on *E. coli* and *M. audouiniiS. africana* on *E. coli, S. aureus* and *M. audouinii* and *K. vesitoria* aerial part on *M. tuberculosis* can be considered significant. The best activity was noted with *S. africana,* with the lowest MIC value of 39.06 μg/ml recorded on three of the nine microorganisms studied. Eight plant extracts found active against *M. smegmatis* were tested against *M. tuberculosis* (Table 2). Interestingly, they were also found to be active against *M. tuberculosis*, the best sample being the extract the aerial part of *K. vesitoria* (MIC of 39.06 μg/ml) and that from the bark of *P. obligun* (MIC of 156.25 μg/ml). However, it has been demonstrated that the sensitivity of *M. tuberculosis* is closer to that of *M. smegmatis*, a non pathogenic microorganism [15]. Therefore, this microorganism can be used for a preliminary study to select samples with potential activity against *M. tuberculosis*[15]. Hence, the results obtained herein are in accordance with such recommendation. Alternative criteria have been described by Fabry *et al.*[28], which consider extracts having MIC values below 8 mg/ml to have noteworthy antimicrobial activity. Under these less stringent criteria, the overall activity recorded with most of the studied extracts can be considered important. However, the tested samples were less active than the RA on all of the microbial strains, this observation been quite normal as it is expected that a crude extract (that contained a panel of both active and non active molecules) shows less activity than a purified compound. The results of the MMC determinations (Table 3) also showed that microbicidal effect of most of the active samples could be expected. The lowest MMC value below 100 μg/ml was recorded with *S. africana*, highlighting the good antimicrobial potency of this plant.

**Table 2:** Minimal inhibition concentration (μg/ml) of the crude extracts and reference antibiotics

**Tested samples**	Tested microorganisms*								
	**Bacteria**					**Fungi**		**Mycobacteria**	
	**βL^+^EC**	**CRCF**	**ARKP**	**CRPA**	**MRSA**	***C. albicans***	***M. audouinii***	***M. smegmatis***	***M. tuberculosis***
*A. oppositifolia* leaves	2500	1250	625	625	312.50	312.50	>2500	>2500	nt
*A. oppositifolia* stem	2500	>2500	>2500	312.50	312.50	312.50	312.50	>2500	nt
*A. karoo* leaves	156.25	312.50	156.25	156.25	156.25	78.12	625	1250	nt
*A. karoo* stem	156.25	312.50	78.12	156.25	78.12	156.25	78.12	1250	2500
*B. latifolia* leaves	2500	2500	>2500	625	1250	625	312.50	>2500	nt
*C. dentata* stem bark	156.25	156.25	156.25	156.25	156.25	78.12	1250	1250	nt
*C. dentate* leaves	1250	625	312.50	312.50	78.12	78.12	>2500	1250	nt
*E. lasianthum* leaves	1250	312.50	156.25	625	312.50	312.50	312.50	>2500	nt
*E. lasianthum* stem	156.25	312.50	312.50	156.25	39.06	78.12	312.50	1250	625
*K. vesitoria* aerial part	2500	2500	>2500	625	>2500	312.50	156.25	1250	39.06
*P. obligum* leaves	625	312.50	312.50	156.25	625	156.25	>2500	>2500	nt
*P. obligun* stem	625	>2500	>2500	312.50	156.25	312.50	312.50	>2500	nt
*P. obligun* bark	78.12	>2500	>2500	>2500	156.25	156.25	78.12	156.25	156.25
*S. burchellii* leaves	312.50	625	156.25	625	312.50	312.50	156.25	>2500	312.50
*S. burchellii* bark	156.25	312.50	156.25	156.25	156.25	156.25	156.25	>2500	nt
*S. hyacithoides* leaf	625	1250	1250	312.50	>2500	312.50	312.50	>2500	nt
*S. hyacithoides* Rhizoma	625	312.50	625	>2500	>2500	156.25	156.25	>2500	nt
*S. africana* aerial part	39.06	312.50	312.50	156.25	39.06	156.25	39.06	312.50	312.50
*T. capensis* aerial part	625	>2500	312.50	625	625	312.50	312.50	>2500	nt
*Z. aethiopica* leaves and stem	625	>2500	>2500	312.50	>2500	312.50	312.50	>2500	nt
Gentamicin	9.76	9.76	19.53	19.53	9.76	nt	nt	nt	nt
Nystatin	nt	nt	nt	nt	nt	19.53	19.53	nt	nt
Ciprofloxacin	nt	nt	nt	nt	nt	nt	nt	0.3	nt
isoniazid	nt	nt	nt	nt	nt	nt	nt	nt	0.13

*The tested microorganisms are MRSA (Methicillin-resistant *Staphylococcus aureus*), βL^+^EC (β-lactamase positive *Escherichia coli*), ARKP (Ampicillin-resistant *Klebsiella pneumoniae*), CRPA (Carbenicillin-resistant *Pseudomonas aeruginosa*), CRCF (Chloramphenicol-resistant *Citrobacter*), *C. albicans* (*Candida albicans*), *M. audouinii* (*Microsporum audouinii*); (nt): not tested.

**Table 3:** Minimal microbicdal concentration (μg/ml) of the crude extracts and reference antibiotics

**Tested samples**	Tested microorganisms*							
	**Bacteria**					**Fungi**		**Mycobacteria**
	**βL^+^EC**	**CRCF**	**ARKP**	**CRPA**	**MRSA**	***C. albicans***	***M. audouinii***	***M. smegmatis***
*A. oppositifolia* leaves	>2500	2500	1250	1250	625	625	nd	>2500
*A. oppositifolia* stem	>2500	nd	nd	1250	625	625	1250	>2500
*A. karoo* leaves	312.50	625	312.50	1250	312.50	312.50	1250	>2500
*A. karoo* stem	625	625	312.50	312.50	156.25	312.50	312.50	>2500
*B. latifolia* leaves	>2500	>2500	nd	1250	2500	1250	625	>2500
*C. dentata* stem bark	312.50	625	312.50	312.50	312.50	312.50	>2500	>2500
*C. dentate* leaves	>2500	2500	625	1250	156.25	156.25	nd	>2500
*E. lasianthum* leaves	2500	625	312.50	1250	625	625	625	>2500
*E. lasianthum* stem	625	625	625	312.50	78.12	312.50	625	>2500
*K. vesitoria* aerial part	>2500	>2500	nd	1250	nd	625	312.50	2500
*P. obligum* leaves	1250	1250	1250	625	1250	312.50	nd	>2500
*P. obligun* stem	1250	nd	nd	625	312.50	625	625	2500
*P. obligun* bark	312.50	nd	nd	nd	625	312.50	312.50	>2500
*S. burchellii* leaves	625	1250	312.50	1250	625	625	312.50	>2500
*S. burchellii* bark	312.50	625	312.50	312.50	312.50	312.50	312.50	>2500
*S. hyacithoides* leaf	1250	2500	2500	1250	nd	625	625	>2500
*S. hyacithoides* Rhizoma	1250	1250	1250	nd	nd	625	625	>2500
*S. fricana* aerial part	78.12	625	625	312.50	78.12	312.50	78.12	2500
*T. capensis* aerial part	2500	nd	1250	1250	1250	1250	625	>2500
*Z. aethiopica* leaves and stem	2500	nd	nd	2500	nd	1250	625	>2500
Gentamicin	19.53	19.53	39.06	39.06	19.53	nt	nt	nt
Nystatin	nt	nt	nt	nt	nt	39.06	39.06	nt
Ciprofloxacin	nt	nt	nt	nt	nt	nt	nt	0.3

*The tested microorganisms are MRSA (Methicillin-resistant *Staphylococcus aureus*), βL^+^EC (β-lactamase positive *Escherichia coli*), ARKP (Ampicillin-resistant *Klebsiella pneumoniae*), CRPA (Carbenicillin-resistant *Pseudomonas aeruginosa*), CRCF (Chloramphenicol-resistant *Citrobacter*), *C. albicans* (*Candida albicans*), *M. audouinii* (*Microsporum audouinii*); (nt): not tested; (nd): not determined as the MIC was >2500 μg/ml.

The results of the present work can be considered important with regards to the medical importance of the studied microorganisms and the fact that most of them were found to be resistant to commonly used antibiotics. *P. aeruginosa* is an important nosocomial pathogen highly resistant to clinically used antibiotics, causing a wide spectrum of infections and leading to substantial morbidity and mortality [29]. Drug-resistant Enterobacteriaceae, including *K. pneumoniaeE. aerogenes* and *E. coli*, have also been classified as antimicrobial-resistant organisms of concern in healthcare facilities [30]. The activity of some of the extracts on *M. smegmatis* also suggested that an inhibition effect of this plant on *M. tuberculosis* could be expected as the sensitivity of this microbial species was found to be closer to *M. smegmatis*, a non pathogenic agent [15], and this hypothesis was confirmed herein.

## Conclusion

The overall results of the present work provide baseline information for the possible use of South African plant extracts in the treatment of microbial infections involving resistant phenotypes.

## Competing interests

The authors declare that they have no competing interests.

## Authors’ contributions

TRHN and VK carried out the study; TRHN, VK, AKJ, JJMM and NL designed the experiments; TRHN and VK wrote the manuscript; All authors read and approved the final manuscript.

## Pre-publication history

The pre-publication history for this paper can be accessed here:

http://www.biomedcentral.com/1472-6882/12/74/prepub
